# The Incorporation of Neurosurgery as an Integral Part of the Strategic Priorities for Surgical Care in Nigeria

**DOI:** 10.3389/fsurg.2021.689180

**Published:** 2021-10-06

**Authors:** Deen L. Garba, Dumura Jeneral Alfin, Muhammad Raji Mahmud

**Affiliations:** ^1^University of North Carolina at Chapel Hill, Chapel Hill, NC, United States; ^2^Department of Global Health and Social Medicine, Harvard Medical School, Boston, MA, United States; ^3^Neurosurgery Division, Department of Surgery, Jos University Teaching Hospital, Katon Rikkos, Nigeria; ^4^Department of Surgery, National Hospital, Abuja, Nigeria

**Keywords:** Nigeria, neurosurgery, global neurosurgery, access, workforce, advocacy

## Introduction

In 2015, the Lancet Commission on Global Surgery estimated nearly 70% of the world still lacked access to safe, affordable emergency and essential Surgical, Obstetrics and Anesthesia (SOA) care, which disproportionately affects those in low- and middle-income countries (LMICs) (1). Pursuant to this landmark study, the World Health Assembly (WHA) passed resolution 68:15 which mandated countries to include emergency and essential surgical, obstetrics and anesthesia care as an integral component of Universal Health Coverage (UHC) (2). This was catalytic in leading Nigeria's Federal Ministry of Health (FMoH) to develop and implement the National Surgical, Obstetrics, Anesthesia and Nursing Plan (NSOANP) in 2017 (3). This plan, dubbed Strategic Priorities for Surgical Care (StraPS), introduced specific surgical system targets and an implementation roadmap that prioritized monitoring, evaluation, and feedback for central and state governments to follow.

Although most Nigerians can reach a healthcare facility within 2 h, the vast majority of these public healthcare centers (aside from the tertiary hospitals) do not have the human resources and infrastructure to provide emergency and essential surgical care when needed. Furthermore, the lack of adequately equipped and fully functional children's hospitals limit the delivery of emergency and essential surgical care for children.

The importance of StraPS was that it afforded children under 15 years old (which constitute 43% of the population in Nigeria) to receive surgery in a surgical plan for the first time while addressing the surgical needs of this unique demographic entity. In addition, StraPS specifically included nursing care, which forms an inseparable component of surgical quality and safety, to ensure nursing is captured in surgical training and workforce development programs.

After brief historical context and an evaluation of the current NSOANP, we provide a brief overview of neurosurgery in Nigeria and current effort toward capacity-building. We then provide a list of recommendations the Nigerian government can adopt in order to improve the quality and delivery of neurosurgical services.

### Neurosurgery and Nigeria's NSOANP

Despite the success of StraPS in prioritizing pediatric surgery and nursing, garnering political support and will for neurosurgical systems strengthening has remained a challenge in Nigeria.

Nigeria has a long tradition of producing excellent neurosurgeons, with the first training program being established in October 1962 under the leadership of the late E. Latunde Odeku (4). Since then, Nigeria has produced several training centers across the country, with close to 100 locally trained neurosurgeons in the last decade as well as the first female neurosurgeon in West Africa. However, lingering challenges which include limited training programs, a dearth of financial investment for educational resources, and insufficiently equipped health facilities continue to create difficulties in accessing care for many patients, which include the prohibitive upfront and catastrophic costs of neurosurgical intervention and follow up care. Therefore, given Nigeria's sizable population, the current workforce remains grossly inadequate despite the laudable recent efforts in increasing the neurosurgical workforce. In addition, this limited, inadequate neurosurgery workforce is largely domiciled within the urban setting, whereas, the large majority of the Nigerian population (60.4%) living in the rural communities are completely deprived of easy access to neurosurgical services (5, 6).

Neurosurgery has been addressed in several contexts in Nigeria's NSOANP, though there are specific areas that require further investigation and investment. For example, the World Health Organization's recommendation is an ideal ratio for every population to be 1 neurosurgeon to 100,000 individuals (7). Within Nigeria's NSOANP, that figure is currently cited to be 0.01 neurosurgeons per 100,000 individuals, which leaves significant room for improvement in order to achieve an acceptable standard of care.

Importantly, the NSOANP does cite neural tube defects as priorities of basic surgical care provision, which is in line with the commonly agreed upon comprehensive spina bifida and hydrocephalus recommendations. Additionally, the intermediate surgical care scope of practice emphasizes the diagnosis and stabilization of neurological trauma such as evacuations for epidural hematoma or emergency burr holes, which is specifically important given the preponderance of traumatic brain injury in LMICs such as Nigeria.

Under the tertiary level scope of practice, the NSOANP also highlights the importance of adequate neuro-anesthesia, which is essential to ensuring neurosurgical intervention can be performed safely and effectively. Neurovascular injuries are also prioritized within this scope, as are congenital anomalies such as meningomyelocele and hydrocephalus, and infections such as intracranial or vertebral osteomyelitis, all suggesting the NSOANP supports these critical elements of neurosurgical care provision.

While the NSOANP makes significant strides in highlighting essential components and the cost necessary for scaling up surgical (including neurosurgical), obstetric, and anesthesia care, it does not detail the specific investments necessary to bring about the desired improvements in prioritizing surgical intervention as well as the larger benefits to society. In other words, there does not appear to be a strategy for funding the plan. For example, per the World Health Organization's recommendations, each nation should dedicate at least 5% of its annual budget to health (7). Presently, per capita public spending for health remains significantly below the US$34 recommended by WHO for low-income countries. A significant increase in investment would be a necessary first step to providing sufficient financial investment for surgical/neurosurgical systems strengthening.

Additionally, plans for scaling up prehospital care which would significantly improve mortality rates from road traffic accidents and time to surgical intervention should be included in a subsequent iteration of this NSOANP. The current literature suggests neurosurgical intervention improves markedly provided timely arrival at a care facility and prompt provision of hemostasis or ICP monitoring, both of which are not specifically mentioned in this current version of the NSOANP. Additional investments such as these will be critical in garnering the necessary political will to invest in neurosurgery and meet both the appropriate workforce demands as well as the harrowing global neurosurgery case deficit, whose burden Nigeria disproportionately suffers from.

### Neurosurgical Recommendation for Consideration in Nigerian's NSOANPS

In the light of this massive neurosurgery care deficit and the enormous challenges that limit the scope of neurosurgical practice within the country, there is a need for a concerted effort aimed at making these services available, accessible and affordable. A deliberate, multipronged approach may be required to address this harrowing issue, which may include the following:

There is an urgent need to include neurosurgical trauma (emergent craniotomies, burr holes and craniectomies) and neural tube anomalies as part of essential and emergency surgical care (i.e., Bellwether procedures) to be performed at minimum of a secondary (intermediate) care level. This is in keeping with the vision of NSOANPS, that is, of a national healthcare system responsive to the surgical needs of all citizens, at all times. This is supported by the fact that neurotrauma cases significantly account for the burden of surgical emergencies in most of the tertiary facilities in Nigeria (3, 6, 8, 9).

The NSOANPS should also consider within its armamentarium the development of a national neurosurgery or trauma care system. This should include, but not limited to, the development of an effective prehospital service system for neurotrauma care, akin to what several nations and regional blocs have recently implemented. In the immediate period, this can be achieved by training of some dedicated members of the available and already existing Federal Road Safety Corp (FRSC) officers and members of the national emergency management agencies (NEMA), to undertake immediate evacuation, Basic Life Support (BLS) resuscitation and transportation of trauma victim, to a nearby facility with capacity for immediate stabilization and resuscitation of such patients. On a long-term basis, a formalized neurotrauma registry should be developed for national monitoring. Such neurotrauma registries allow for simple demographic and case-specific information to be collated within a database for data, thereby allowing for tracking of disease pathologies, a reduction of delays to surgery and overall improved patient outcomes. Furthermore, the development of these registries could bolster other national health priorities, by being repurposed toward enhancing performance improvement processes through outcomes evaluation and allowing for streamlined provider credentialing, accreditation, and verification. In tandem, additional structures can be put in place to encourage private sector participation in prehospital emergency service delivery and transportation. This will encourage and strengthen the implementation of existing national ambulance policy, as suggested in the NSOANPS documentation (3).

As part of an effective approach to neurosurgery access, the Strategic Priorities for Surgical Care (StraPS) of the Federal Ministry of Health (FMoH) should consider the development of an appropriate referral, transport and feedback system, between hospitals with and those without neurosurgery services. Such a strategy will require a multi-disciplinary team approach, with well-defined roles and responsibilities and a proper communication network system. This approach will help eliminate the common yet erroneous perception that, surgery frequently consists of a surgeon and an anesthetist in a sterile environment. However, a more accurate perspective acknowledges an interdependent network of individuals and institutions all essential to the delivery of safe, timely, and affordable surgical and anesthesia care. Many of these components are not standalone requirements for a surgical system, but rather for a shared delivery infrastructure that is the basis of a functional health system.

There is an urgent need to address the scope and challenges of the neurosurgical work force deficit in Nigeria, which NSOANPS currently captures as 0.01 neurosurgeons per 100,000 individuals (3). A two-tiered approach to training can be adopted in order to address the immediate need while working on the long term strategy to improve the number of trained neurosurgeons in the country. For example, a fast – tracked, competency-based certification of General and Pediatric surgeons who are capable of performing neurotrauma operations and neural tube defect surgeries could be adopted. Secondly, the acceleration of neurosurgical training by both the National Post graduate Medical College of Nigeria (NPMCN) and West African College of Surgeon (WACS), through the promotion and retention of neurosurgery faculty and the establishment of an appropriate curriculum that allows interested candidates the opportunity to proceed immediately into neurosurgery training without going through a general surgery training. This approach was also recommended by Parks et al. in “addressing the problem of restricted access to neurosurgical care in rural sub-Saharan Africa” (10). Accordingly, novel training models could also be adopted, especially those aimed at improving the quality of neurosurgical education via electronic video or zoom conferences at partner institutions. Such platforms, examples of which include Proximie, OssoVR, and PrecisionOS, allow trainees to discuss complex cases simultaneously with other centers worldwide and should be incorporated into neurosurgery training programs (11).

The establishment of twinning programs with partner facilities and institutions in high income countries (HICs) for collaborative training and research opportunities has also been demonstrated to improve access to quality and safe neurosurgical service, and could be a priority for Nigeria (12). These novel paradigms, such as the International Neurosurgery Twinning Initiative Modeled for Africa (INTIMA), apply a multi-phased approach in resolving identified gaps in neurosurgical infrastructure and care pathway that will sustain or deepen the dimensions of unmet neurosurgery in a given local environment (13, 14). Moreover, there is a need to actively encourage, recruit and retain students and young physicians interested in neurosurgical practice into the field and also retain the existing neurosurgeons by preventing further “brain-drain” within the country, consistent with previous trends (10, 15).

### Financing for Essential Neurosurgical Services

Surgical care financing in Nigeria follows the same trend as the financing of healthcare costs in the country, which remains suboptimal. Furthermore, due to the abysmal funding of health care services by the federal government budget in Nigeria, patient's out of pocket expenditures have been shown to be around 73%, yet this remains the most common source of financing health care cost in the country. Other means include public spending on healthcare (12%), health insurance (<3%) and the rest being donor funding. Neurosurgical services within Nigeria remain costly due to the high costs of manpower training and equipment, and accessing such services by households to cover surgical care frequently results in catastrophic expenditure. Therefore, the inclusion of all essential neurosurgical services (operative and non-operative) into the National Health Insurance Services (NHIS) scheme, in addition to the numerous recommendations for the financing of surgical care outlined in the NSOANPS document should be given serious consideration by government and policy makers. This will help bridge the gaps in accessing timely, affordable, quality and safe neurosurgical care (3).

### The Importance of Neurosurgical Infrastructure

For a health care system to meet the surgical needs of the population there is also a need for appropriate infrastructure and equipment to support the human resources in delivering efficient health care and surgical services, which is equally important for neurosurgical services in Nigeria. Therefore, the establishment of efficient and effective procurement and logistics management systems is required. This helps to ensure the ongoing availability of medical and neurosurgical supplies and consumables at all levels of neurosurgery care.

Additional strategies, such as the provision of functional, up-to-date, affordable, high quality and durable equipment through an efficient import system, private public partnerships and international collaboration with agencies or hospitals that are willing to supply or donate equipment to developing countries, should be employed as well. Sustainable local production of all or parts of the necessary equipment should be encouraged, as should adequate training of biomedical engineers and technicians to manage and maintain the appropriate neurosurgical infrastructure. Planned regular maintenance and calibration of equipment, with up-to-date procurement or access to spare parts through maintenance and supply contracts with manufacturer services should also be put in place. Periodic audit of available and functional equipment and provision of back-up mechanisms, to ensure compliance with accepted international safe standards should be promoted.

The promotion of neurosurgery research, through national and international collaboration is an area largely neglected by most authors advocating for improved, affordable and accessible neurosurgical services in developing countries and Nigeria. The NSOANPS policy document laid little or no emphasis on research as an important aspect of improving surgical care. High-quality medical research can guide clinical practice, inform health policy formulation and implementation. Importantly, neurosurgery research was seen as an important recommendation for improving and expanding neurosurgery services by some authors (16–20).

## Conclusion

The engagement of the neurosurgical community with health care planners and policy makers to establish and acknowledge the value of providing neurosurgical care in their countries was recently codified in the Bogota Declaration, signed by eminent members of the global neurosurgical community and endorsed by a representative of the WHO in late 2016 (15, 21).

The advocacy and sensitization of the Nigerian government and policy makers on the importance and need for improved, safe neurosurgery services that are easily accessible and affordable should be the driving force behind achieving the Strategic Priorities for Surgical Care (StraPS) with respect to neurosurgery in Nigeria. Such advocacy will also necessarily involve hospital administrators and end users as well. The neurosurgery community within the country needs to advocate for mentorship and training of its members as to how to achieve self-determination, self-improvement and self-motivation, all of which are key strategies to providing comprehensive and effective neurosurgical services, in addition to the strategies outlined above toward improving the provision of neurosurgical care in Nigeria.

## Author Contributions

All authors listed have made a substantial, direct and intellectual contribution to the work, and approved it for publication.

## Conflict of Interest

The authors declare that the research was conducted in the absence of any commercial or financial relationships that could be construed as a potential conflict of interest.

## Publisher's Note

All claims expressed in this article are solely those of the authors and do not necessarily represent those of their affiliated organizations, or those of the publisher, the editors and the reviewers. Any product that may be evaluated in this article, or claim that may be made by its manufacturer, is not guaranteed or endorsed by the publisher.
